# Comparative Effectiveness of Calcium‐Channel Blockers, Angiotensin‐Converting Enzyme/Angiotensin Receptor Blockers and Diuretics on Cardiovascular Events Likelihood in Hypertensive African‐American and Non‐Hispanic Caucasians: A Retrospective Study Across HCA Healthcare

**DOI:** 10.1002/clc.70075

**Published:** 2025-01-21

**Authors:** Anil Harrison, Sushil Rayamajhi, Farhad Shaker, Schwartz Thais, Melissa Moreno, Kaveh Hosseini

**Affiliations:** ^1^ Department of Medicine Midwestern University Glendale Arizona USA; ^2^ Department of Internal Medicine University of Central Florida College of Medicine/HCA Florida West Hospital Pensacola Florida USA; ^3^ Tehran Heart Center, Cardiovascular Disease Research Institute Tehran University of Medical Sciences (TUMS) Tehran Iran; ^4^ Department of Research and Statistics HCA Healthcare Research Nashville Tennessee USA; ^5^ Department of Mathematics and Systems Engineering Florida Institute of Technology Melbourne Florida USA

**Keywords:** African American, antihypertensive agents, cardiovascular events, hypertension, non‐Hispanic Caucasians

## Abstract

**Background:**

Hypertension, a leading global risk factor for mortality and disability, disproportionately affects racial and ethnic minorities. Our study investigates the association between the type of prior antihypertensive medication use and the likelihood of cardiovascular events (CVE) and assesses whether the patient's race influences this relationship.

**Methods:**

A retrospective study of 14 836 hypertension cases aged ≥ 40 years was conducted using data from HCA Healthcare between 2017 and 2023. Logistic regression was employed to predict the likelihood of CVE and mortality at admission, adjusting for baseline comorbidities, with Race added as an effect modifier. Interaction analysis was performed among races based on antihypertensive medication types.

**Results:**

African American patients on ACE inhibitors (ACE) or angiotensin receptor blockers (ARBs) were 1.7 times more likely to have cardiovascular events (CVE) compared to those on calcium channel blockers (CCBs) and 0.66 times as likely compared to diuretics. CCB users had a lower CVE risk than diuretic users. Among White patients, ACE/ARB users had a 1.18 times higher CVE risk than CCB users and 0.45 times lower compared to diuretics, while CCBs offered a 0.38 times lower risk than diuretics. Only ACE/ARB use showed significantly higher CVE odds for African Americans compared to White patients, with similar risks across racial groups for CCBs and diuretics.

**Conclusion:**

Prior antihypertensive type significantly influenced CVE risk, with race as an effect modifier. CCB users had lower CVE odds than ACE/ARBs or diuretics, and ACE/ARBs showed reduced CVE likelihood compared to diuretics in both racial groups.

## 1 Introduction

Hypertension is the most prevalent risk factor for both mortality and disability‐adjusted life‐years worldwide [1, 2]. Cardiovascular events remain the primary cause of death globally and are responsible for almost 18 million deaths annually, which constitutes about one‐third of all deaths worldwide [2, 3]. Hypertension is a significant modifiable risk factor for cardiovascular events [4]. The introduction of the American College of Cardiology and the American Heart Association hypertension guidelines in 2017 resulted in an increase in the estimated number of individuals diagnosed with hypertension [5, 6]. Despite varying diagnostic criteria for hypertension, the treatment recommendations remain similar and are primarily based on the risk of cardiovascular events [7, 8].

Despite efforts to enhance blood pressure (BP) control and management, hypertension management has not been equitable among racial and ethnic minority groups [9]. An analysis of hypertension control rates from the National Health and Nutrition Examination Survey (NHANES) revealed that BP control was achieved less frequently among Hispanic individuals (40%), non‐Hispanic (NH) Black individuals (39%), and Asian American individuals (38%) compared to NH White individuals (49%) [9]. Furthermore, NH Black individuals are diagnosed with hypertension earlier and suffer from higher hypertension‐related morbidity and mortality than NH White individuals. They face a 30% higher risk of fatal stroke and a 50% higher risk of cardiovascular disease (CVD) mortality [10]. Overall, NH Black individuals experience hypertension‐related mortality rates that are four to five times higher than those of NH White Americans [11]. Additionally, NH Black adults have lower hypertension control rates and less treatment intensification [11].

The 2017 American College of Cardiology (ACC)/American Heart Association (AHA) blood pressure guideline recommends calcium channel blockers (CCBs) and thiazide diuretics as first‐line agents for non‐Hispanic (NH) Black adults with hypertension who do not have heart failure or renal disease [11]. The guideline also emphasizes the use of antihypertensive medications from two or more different classes [11]. Conversely, the International Society of Hypertension in Blacks (ISHIB) advises combination therapy with a CCB and a renin‐angiotensin blocking agent (preferably an angiotensin receptor blocker [ARB]) as the primary treatment for NH Black patients with hypertension [12]. Numerous trials have assessed the efficacy of different antihypertensive regimens, yielding conflicting results, particularly in racial and ethnic‐specific studies. The Antihypertensive and Lipid‐Lowering Treatment to Prevent Heart Attack Trial (ALLHAT) found that angiotensin‐converting enzyme (ACE) inhibitors, CCBs, and alpha‐receptor blockers were not superior to thiazide diuretics in lowering blood pressure [12]. Thiazide diuretics were also more effective in preventing strokes among NH Black individuals compared to other medications [12]. However, the African American Study of Kidney Disease indicated that ACE inhibitors were more effective than beta‐blockers and CCBs in slowing the progression of renal disease in NH Black Americans with nondiabetic renal disease, contradicting ALLHAT's findings that ACE inhibitors were not superior to other antihypertensive classes for preventing kidney disease [12, 13].

Understanding the optimal first‐line antihypertensive medications for preventing cardiovascular events and mortality is crucial for clinical decision‐making. Previous studies have explored the efficacy of antihypertensive treatments in reducing cardiovascular events [14, 15, 16]. Identifying the most effective treatments for controlling hypertension and reducing cardiovascular events and mortality with minimal side effects is essential for guiding clinicians and reducing the global burden of cardiovascular disease. To provide an updated perspective on the comparative efficacy of antihypertensive medications, we aimed to investigate the association between prior antihypertensive medication use and the likelihood of cardiovascular events and to assess whether this relationship is influenced by the patient's race. The findings from this study will be relevant for contemporary clinical management of hypertension, especially considering the new ACC/AHA hypertension guidelines [8].

## 2 Methods

In this retrospective study, we focused on a population residing in the Far West region from a divisional database of patients admitted to HCA Healthcare from January 1, 2017, to July 1, 2023. The study was approved, and data were cleared by the HCA Institutional Review Board, which waived informed consent for this retrospective, deidentified data set because of minimal risk to patients.

### 2.1 Study Setting, Inclusion, and Exclusion Criteria

We used data from HCA Healthcare, a large and diverse healthcare system serving the Far West region of the USA serving metropolitan, urban, and rural populations. Individuals aged over 40 years and diagnosed with hypertension were included in this study. Additionally, participants were required to have been taking at least one antihypertensive medication before admission. Only non‐Hispanic White or Black patients were considered eligible for inclusion. Exclusion criteria were applied to patients diagnosed with atrial fibrillation or with a history of A‐fib, those with known drug use, individuals who were HIV positive, or those with hyperviscosity syndromes. Pregnant patients were also excluded from the study. Furthermore, patients taking more than one antihypertensive medication before admission, except for ACE or ARBs, were excluded. These criteria were employed to ensure a homogeneous study population and maintain the research findings’ integrity and relevance.

These cases were investigated to determine whether or not they had experienced a cardiovascular event. A cardiovascular event refers to any incident that may cause damage to the heart or blood vessels, which can significantly impact cardiovascular health. These events are typically acute and can be life‐threatening. Common types of cardiovascular events include Myocardial Infarction, Stroke, Heart Failure, Arrhythmia, Peripheral Artery Disease (PAD), and so on. These events are critical concerns due to their high morbidity and mortality rates, and they often require immediate medical attention and intervention to prevent long‐term damage or death.

### 2.2 Variables

Age was recorded as a numeric variable representing the patient's age in years. Sex was a binary variable with values for females and males. Race was categorized into African American/Black and White. Smoking status was a binary variable with smokers and non‐smokers. Cardiovascular Events were binary variables indicating the presence or absence of these conditions, with specific diagnosis codes provided. Diabetes and Chronic Kidney Disease (CKD) were binary variables indicating whether a diagnosis was present or absent. Systolic and diastolic blood pressure readings were binary variables, indicating normal ( ≤ 140 mmHg for systolic and ≤ 90 mmHg for diastolic) or high readings ( > 140 mmHg for systolic and > 90 mmHg for diastolic). Prior admission medications were categorized into ACE/ARBs, Calcium Channel Blockers, and Diuretics.

### 2.3 Statistical Analysis

A binary logistic regression analysis was conducted to predict the likelihood of cardiovascular events at index admission, with antihypertensive medication taken before admission as the independent variable and race as an effect modifier. Race was added as an effect modifier to assess whether there was an association between the outcome of the use of each antihypertensive class before admission and race. The model incorporated the race interaction with antihypertensive medication as the primary exposure variable while adjusting for age, smoking status, statin, aspirin use, diabetes, and chronic kidney disease. An analysis of interaction using Least Squares Means with a Tukey‐Kramer adjustment for multiple comparisons was conducted to determine where there were differences within and between the races based on the antihypertensive medication used before admission. Analyses were conducted using SAS software (SAS Inc, Cary, North Carolina), and P‐values less than 0.05 were considered statistically significant.

## 3 Results

### 3.1 Study Population

Initially, the population of individuals aged over 40 years diagnosed with hypertension and experiencing either a stroke/cerebral event or a cardiovascular event admitted to HCA Healthcare during the period from January 1, 2017, to July 1, 2023, totaled 43 700 individuals. Excluding patients from this initial pool due to leaving against medical advice, being discharged to a psychiatric hospital, and missing race and/or ethnicity data left a remaining population of 37 010 individuals.

**Figure 1:**
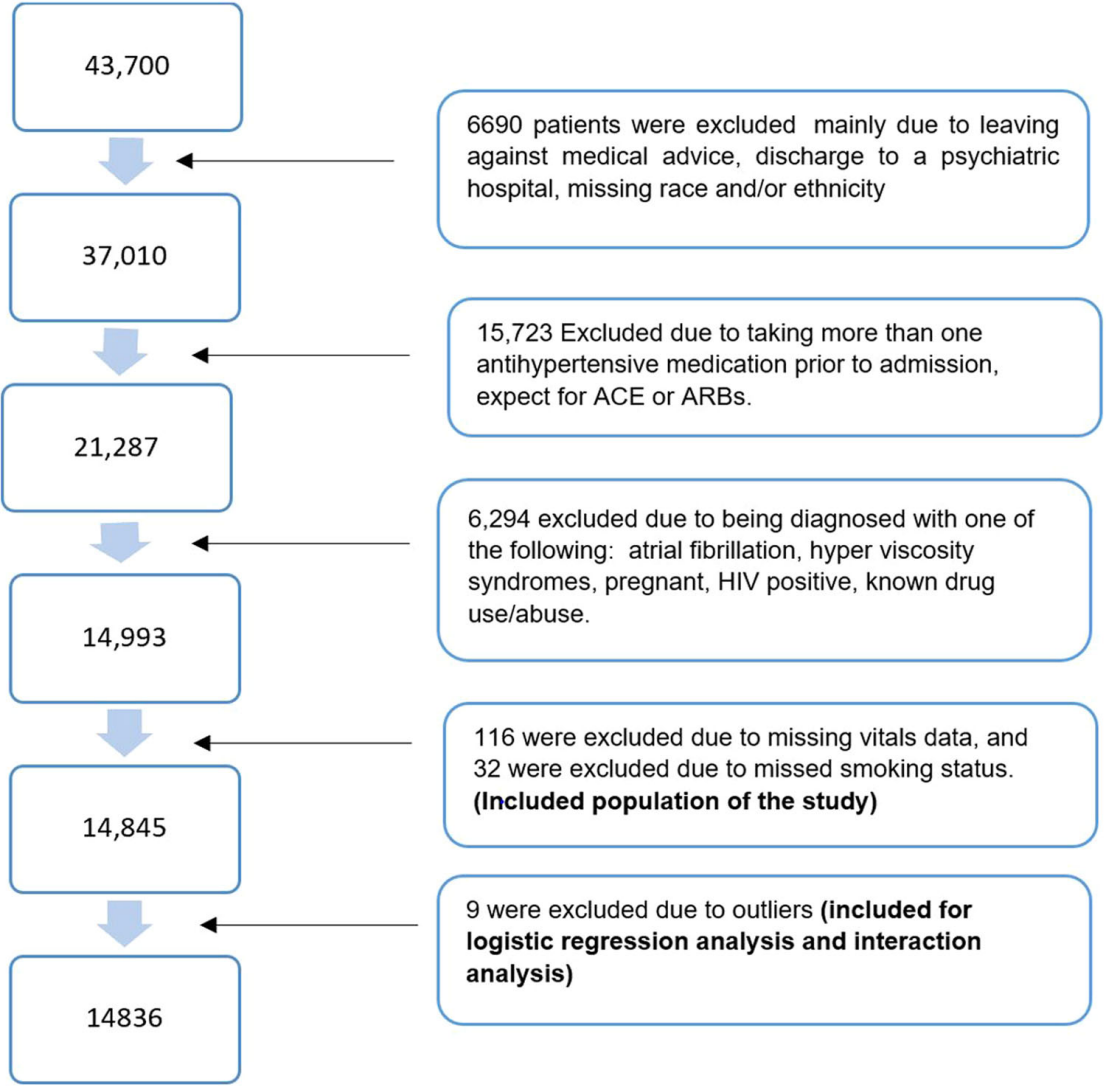
**Flowchart of population inclusion/exclusion process**.

Further exclusions were made among this group. Specifically, individuals taking more than one antihypertensive medication before admission, except for ACE inhibitors or ARBs, were excluded, resulting in 21 287 remaining individuals. Among these, 6294 individuals were excluded due to specific diagnoses, including atrial fibrillation, hyperviscosity syndromes, pregnancy, HIV positivity, or known drug use/abuse, leaving a population of 14 993 individuals. Subsequently, individuals without smoking status data and missing vital signs were excluded, resulting in a final study population of 14 845 individuals, of which nine patients were excluded for logistic regression and interaction analyses as outliers, and a total of 14 836 cases were included in our analyses. Figure 1 outlines the process of selecting the study population, providing detailed numbers and percentages at each stage.

### 3.2 Description of Study Population

Among the 14 845 individuals included in the analysis, 22.9% were identified as Black, while 77.1% were categorized as White Caucasian, representing the racial composition of the cohort. Regarding sex distribution, 54.4% of Black individuals and 52.5% of White Caucasians identified as female, with the overall proportion being 52.9%. Analysis of admission sources revealed that 95.0% of Black individuals and 94.2% of White Caucasians were admitted from their homes, with a slight variation in percentages observed overall (94.4%). Discharge disposition indicated that 78.5% of Black individuals and 76.8% of White Caucasians were discharged home, with an overall proportion of 77.2%.

Further examination of the study population's characteristics revealed variations in smoking status and insurance coverage. Notably, 78.6% of Black individuals and 79.4% of White Caucasians were nonsmokers, while 21.4% of Black individuals and 20.6% of White Caucasians reported smoking. Insurance coverage predominantly consisted of MEDICARE/CAID, with 78.2% of Black individuals, 80.0% of White Caucasians, and 79.6% overall enrolled in this category. Income distribution revealed disparities, with 20.6% of Black individuals, 24.6% of White Caucasians, and 23.7% overall falling within the income bracket of $35 000–54 999. Notably, a higher percentage of White Caucasians fell into the income bracket of 55 000–89 999 compared to Black individuals.

Diabetes, a common chronic condition, was more prevalent among Black individuals, with 56.3% affected, compared to 44.0% of Caucasian individuals. Table 1 illustrates the baseline demographic and clinical characteristics of both racial subgroups.

**Table 1:** Characteristics of study population.

Variables	Black		White Caucasian		Overall	
	*n*	%	*n*	%	*n*	%
*n*, %	3404	22.9%	11 441	77.1	14 845	100.0
**Sex**	1852	54.4	6005	52.5	7857	52.9
F						
M	1552	45.6	5436	47.5	6988	47.1
**Admission source**	3234	95.0	10 777	94.2	14 011	94.4
Home						
Hospital	149	4.4	587	5.1	736	5.0
Unknown	21	0.6	77	0.7	98	0.7
**Discharge disposition**	2671	78.5	8792	76.8	11 463	77.2
Discharged Home						
Expired	50	1.5	106	0.9	156	1.1
Hospice	74	2.2	355	3.1	429	2.9
Transferred to Hospital	54	1.6	215	1.9	269	1.8
Transferred to SNF/LTCH/Rehab	554	16.3	1971	17.2	2525	17.0
**Smoking status**	2675	78.6	9084	79.4	11 759	79.2
No						
Yes	729	21.4	2,357	20.6	3086	20.8
**Insurance type**	174	5.1	585	5.1	759	5.1
Gov						
MEDICARE/CAID	2662	78.2	9,158	80.0	11 820	79.6
No Insurance	142	4.2	358	3.1	500	3.4
Other	72	2.1	181	1.6	253	1.7
Private Insurance	354	10.4	1159	10.1	1513	10.2
**Income**	702	20.6	2809	24.6	3511	23.7
35 000–54 999						
55 000–89 999	222	6.5	2473	21.6	2695	18.2
Greater than 90 000	16	0.5	145	1.3	161	1.1
Less than 35 000	788	23.1	913	8.0	1701	11.5
Unknown	1676	49.2	5101	44.6	6777	45.7
**Diabetes**	1918	56.3	5033	44.0	6951	46.8
**CKD**	1541	45.3	3755	32.8	5296	35.7
**Variables**	**M**	**SD**	**M**	**SD**	**M**	**SD**
Age	64	12	70	12	68	12
Max Systolic Vital (avg)	162.5	22.1	157.5	22.3	158.6	22.4
Max Diastolic Vital (avg)	89.9	12.9	84.6	12.1	85.8	12.5

### 3.3 Medications Prior to Admission

Supporting Information S1: Table S1 delineates the medication profile of the study cohort before admission, dissected by racial demographics and overall figures. Statins, employed primarily for cholesterol management, were prescribed to 40.0% of Black individuals and 46.5% of Caucasians, constituting 45.0% of the total population. Similarly, aspirin, a common antithrombotic agent, was utilized by 29.4% of Black individuals, 31.5% of Caucasians, and 31.1% overall. Notably, ACE inhibitors or angiotensin receptor blockers (ACE/ARB) seemed to be more frequently prescribed to Caucasians, with 67.1% utilizing them compared to 57.7% of Black individuals, representing 65.0% of the total population. Calcium channel blockers exhibited a numerically higher usage rate among Black individuals compared to Caucasians (28.5% vs. 18.3%, respectively), while diuretics were utilized by 13.8% of Blacks versus 14.6% of Caucasians.

Renin‐inhibitors were not utilized by Black individuals, and their overall usage was negligible. Beta‐blockers, alpha‐blockers, and vasodilators displayed comparable patterns across racial groups, albeit with varying utilization rates.

## 4 Outcomes

Among the total cohort of 14 845 individuals, mortality rates differed between the two racial groups, with 1.5% of Black individuals and 0.9% of Caucasian individuals experiencing mortality, constituting 1.1% of the overall population. Cardiovascular events were more prevalent among Black individuals, with 40.2% experiencing such events, compared to 30.7% of Caucasian individuals. Additionally, stroke or cerebral events occurred in 7.3% of Black individuals and 5.9% of Caucasian individuals.

Hospice referral rates also exhibited disparities, with 2.2% of Black individuals referred to hospice compared to 3.1% of Caucasian individuals. Moreover, ICU admissions, indicative of severe illness, were more prevalent among Black individuals, with 16.4% admitted, compared to 14.8% of Caucasian individuals.

Black individuals exhibited a higher mean LOS of 5.9 days compared to Caucasian individuals, who had a mean LOS of 5.1 days. The mean ICU LOS for Black individuals was 4.0 days, despite 3.3 days for Caucasians (Table 2).

**Table 2:** Outcomes summary.

Variables	Black		Caucasian		Overall	
	*n*	%	*n*	%	*n*	%
*n*, %	3404	22.9	11 441	77.1	14 845	100.0
Mortality	50	1.5	106	0.9	156	1.1
Hospice Referral	74	2.2	355	3.1	429	2.9
Mortality/Hospice	124	3.6	461	4.0	585	3.9
ICU	557	16.4	1691	14.8	2248	15.1
ED	2840	83.4	8781	76.8	11 621	78.3
Stroke or Cerebral Event	247	7.3	673	5.9	920	6.2
Cardiovascular Event	1369	40.2	3515	30.7	4884	32.9
**Variables**	**M**	**SD**	**M**	**SD**	**M**	**SD**
LOS	5.9	10.2	5.1	5.5	5.3	6.9
ICU LOS	4.0	5.5	3.3	3.7	3.5	4.2

### 4.1 Associated Factors to Cardiovascular Events

A binary logistic regression analysis on a sample of 14 836 patients. Race was added as an effect modifier to assess whether there was an association between the antihypertensive used before admission and race. The interaction or effect modifier of race and antihypertensive used was found to be statistically significant (*p* < 0.0001). The relationship between the type of antihypertensive used before admission and a diagnosis of cardiovascular events varied depending on the race of the patient. Thus, we included this interaction term in the final model. The c‐statistic for the model was approximately 0.66.

The model incorporated the race interaction with antihypertensive medication as the primary exposure variable while adjusting for age, smoking status, statin, aspirin use, diabetes, and chronic kidney disease. Diabetes, chronic kidney disease (CKD), smoking status, and aspirin usage before admission were all significantly associated with the likelihood of being diagnosed with a cardiovascular event after controlling for other variables. All other variables held constant; those diagnosed with diabetes were 1.184 times as likely to be diagnosed with a cardiovascular event as compared to those not diagnosed with diabetes (*p* < 0.0001, 95% CI [1.099, 1.275]), those diagnosed with CKD were 1.933 times as likely to be diagnosed with a cardiovascular event as compared to those not diagnosed with CKD (*p*‐value < 0.0001, 95% CI [1.794, 2.082]), smokers were 1.325 times as likely to be diagnosed with a cardiovascular event as compared to non‐smokers (*p* < 0.0001, 95% CI [1.209, 1.452]), and those who were taking aspirin before admission were 1.608 times as likely to be diagnosed with a cardiovascular event when compared to those not taking aspirin (*p* < 0.0001, 95% CI [1.488, 1.738]) (Table 3).

**Table 3:** Analysis of penalized maximum likelihood estimates.

Parameter		DF	Estimate	Standard	Wald			95% CI OR	
				Error	Chi‐Square	Pr > Chi Sq	OR	Lower	Upper
Intercept		1	‐1.237	0.127	94.901	< 0.0001			
**Age**		1	0.008	0.002	26.167	< 0.0001	1.008	1.005	1.012
Race		1			40.053	< 0.0001			
**Diabetes**	1 versus 0	1	0.169	0.038	19.831	< 0.0001	1.184	1.099	1.275
**CKD**	1 versus 0	1	0.659	0.038	300.012	< 0.0001	1.933	1.794	2.082
**Smoker**	1 versus 0	1	0.281	0.047	36.062	< 0.0001	1.325	1.209	1.452
Statin	1 versus 0	1	0.041	0.038	1.167	0.280	1.042	0.967	1.123
**Aspirin**	1 versus 0		0.475	0.040	142.842	< 0.0001	1.608	1.488	1.738
Hypertensive Meds		2			192.756	< 0.0001			
Race*Hypertensive		2			18.679	< 0.0001			

### 4.2 Model Fit

The model fit statistics indicate the adequacy of the logistic regression model in predicting cardiovascular events among the study population. The c‐statistic, a measure of discrimination, was approximately 0.66, suggesting that the model demonstrates good discriminatory ability. Additionally, the Hosmer and Lemeshow goodness‐of‐fit test yielded a p‐value of 0.1039, indicating that the model fits the data adequately. Furthermore, the association between predicted probabilities and observed responses was evaluated using other metrics. The percent concordant, which measures the agreement between predicted probabilities and observed outcomes, was found to be 65.9%. Somers’ D, another measure of predictive accuracy, was calculated at 0.319. Additionally, the percent discordant was 34.0%, indicating some disagreement between predicted probabilities and observed responses. The gamma coefficient, a measure of the strength and direction of association between predicted probabilities and observed responses, was also found to be 0.319. Moreover, the percent tied, representing the proportion of observations where the predicted probabilities are equal, was determined to be 0.0%. Finally, the Tau‐a coefficient, which evaluates the degree of association between predicted probabilities and observed outcomes, was calculated at 0.141 (Supporting Information S1: Table S2).

Overall, these model fit statistics suggest that the logistic regression model adequately predicts cardiovascular events in the study population, with the c‐statistic, percent concordant, Somers’ D, and other metrics indicating reasonable predictive accuracy and discrimination ability.

### 4.3 Analysis of Interaction

As mentioned before, among patients with hypertension, the type of medication used before admission was significantly associated with the likelihood of being diagnosed with a cardiovascular event after controlling for other variables. This relationship varied depending on the patient's race (Table 4).

**Table 4:** Differences of antihypertensive meds least squares means.

Adjustment for Multiple Comparisons: Tukey‐Kramer										
									95% CI OR	
Comp	Comp	Ref	Ref	Estimate	Standard error	z Value	Adj P	Odds ratio	Adj lower	Adj upper
**Black**	**ACE/ARBs**	**Black**	**Calcium B**	0.5309	0.08579	6.19	< 0.0001	1.700	1.332	2.171
**Black**	**ACE/ARBs**	**Black**	**Diuretics**	‐0.4186	0.1059	‐3.95	0.0011	0.658	0.487	0.890
**Black**	**ACE/ARBs**	**White**	**ACE/ARBs**	0.5728	0.05458	10.49	< 0.0001	1.773	1.518	2.072
**Black**	**ACE/ARSs**	**White**	**Calcium B**	0.7408	0.07057	10.5	< 0.0001	2.098	1.715	2.565
**Black**	**ACE/ARBs**	**White**	**Diuretics**	‐0.2365	0.06936	‐3.41	0.0085	0.789	0.648	0.962
**Black**	**Calcium B**	**Black**	**Diuretics**	‐0.9495	0.1189	‐7.98	< 0.0001	0.387	0.276	0.543
Black	**Calcium B**	White	ACE/ARBSs	0.04187	0.07726	0.54	0.9944	1.043	0.837	1.300
Black	**Calcium B**	White	**Calcium B**	0.2099	0.08903	2.36	0.1715	1.234	0.957	1.590
**Black**	**Calcium B**	**White**	**Diuretics**	‐0.7674	0.08814	‐8.71	< 0.0001	0.464	0.361	0.597
**Black**	**Diuretics**	**White**	**ACE/ARBs**	0.9913	0.0991	10	< 0.0001	2.695	2.032	3.574
**Black**	**Diuretics**	**White**	**Calcium B**	1.1593	0.1086	10.67	< 0.0001	3.188	2.339	4.344
Black	Diuretics	White	Diuretics	0.1821	0.1078	1.69	0.5391	1.200	0.882	1.631
**White**	**ACE/ARSs**	**White**	**Calcium B**	0.168	0.05759	2.92	0.0412	1.183	1.004	1.394
**White**	**ACE/ARBs**	**White**	**Diuretics**	‐0.8092	0.05678	‐14.25	< 0.0001	0.445	0.379	0.523
**White**	**Calcium B**	**White**	**Diuretics**	‐0.9772	0.072	‐13.57	< 0.0001	0.376	0.307	0.462

Regarding patients self‐identifying as African American/those who were taking ACE or ARBs before admission were 1.700 times as likely to be diagnosed with a CVE as those who were taking calcium channel blockers (*p*‐value < 0.0001, 95% CI [1.332, 2.171]). In addition, those who were taking ACE or ARBs before admission were 0.658 times as likely to be diagnosed with a CVE compared to those who were taking diuretics (*p*‐value = 0.0011, 95% CI [0.487, 0.890]). Finally, those taking calcium channel blockers before admission were 0.387 times as likely to be diagnosed with a CVE compared to those who were taking diuretics (*p*‐value < 0.001, 95% CI [0.276, 0.543]).

Concerning patients self‐identifying as non‐Hispanic White by the type of medication used before admission, a similar relationship as observed in the African American/Black group was observed. Patients who self‐identified as White and were taking ACE or ARBs before admission were 1.183 times as likely to be diagnosed with a cardiovascular event compared to Whites who were taking calcium channel blockers before admission (*p*‐value = 0.0412, 95% CI [1.004, 1.394]). While those taking ACE or ARBs before admission were 0.445 times as likely to be diagnosed with a cardiovascular event as compared to those who were taking diuretics before admission (*p*‐value < 0.0001, 95% CI [0.379, 0.523]), those who were taking calcium channel blockers before admission were 0.376 times as likely to be diagnosed with a cardiovascular event as compared to those who were taking diuretics before admission (*p*‐value < 0.0001, 95% CI [0.307, 0.462]).

Finally, we compared those patients self‐identifying as African American/Black to those self‐identifying as non‐Hispanic White to each other based on the type of medication used before admission. In comparing all patients taking ACE or ARBs by race, all patients taking calcium channel blockers by race, and all patients taking diuretics by race, only the ACE or ARBs group showed a statistically significant difference, as among those taking ACE/ARBs before admission who self‐identified as Black were 1.773 times as likely to be diagnosed with a cardiovascular event when compared to those self‐identifying as non‐Hispanic White.

## 5 Discussions

Our study demonstrated a significant interaction between the race of the patient and the type of antihypertension used before admission and the diagnosis of CVE, highlighting the importance of considering race in determining the management plans of HTN. In addition, both White and Black subgroups being treated with CCBs were shown to have lower odds of being diagnosed with CVE compared to ACE/ARBs and Diuretics. Furthermore, as an interesting finding in both subgroups, ACE/ARBs were superior to diuretics in terms of CVE diagnosis likelihood in both racial subgroups. While CCBs and diuretics showed comparable efficacy between racial subgroups, Black cases using ACE/ARB demonstrated a significantly higher rate of CVE compared to Whites.

Considering the consumption frequency of each antihypertension drug class, ACE/ARBs were the most used drug by both Blacks (57.7%) and NHC (67.1%). All investigated demographic baseline features were similar between racial subgroups except for income, which was higher among Whites. Regarding baseline clinical data and outcomes, an overall higher rate of DM and CKD was observed in Blacks, as well as a higher rate of CVE, mortality, ICU admission, and LOS.

Our logistic regression model, which demonstrated an acceptable accuracy and discriminating efficacy (c‐statistic of 0.66), demonstrated a significant association between the antihypertensive used before admission and a diagnosis of CVE by race. In addition, age, DM, CKD, smoking history, and using aspirin Were independently associated with CVE, while the history of using statin was not. Our findings are in line with Clemmer et al. study on almost 16 000 HTN patients, which revealed a significant association between older age, Black race, higher BMI, and worse response to antihypertensive agents [17]. These findings underscore the importance of considering race as a potential effect modifier in the relationship between antihypertensive medication use and cardiovascular outcomes. Further exploration of these interactions is crucial for developing personalized approaches to hypertension management and reducing disparities in cardiovascular health outcomes among diverse patient populations.

Our study showed lower odds of being diagnosed with CVE in patients using CCBs compared to ACE/ARBs in both Black and non‐Hispanic Caucasian patient subgroups. ALLHAT trial reported a numerically higher rate of cardiovascular disease in patients using ACEi compared to the CCBs [12, 18]; however, this difference was more prominent in the Black subgroup, which is similar to our finding. In addition, a multi‐center retrospective large‐population study demonstrated a higher risk of composite adverse cardiac outcome in HTN Black patients treated with ACEi compared to CCBs with a Hazard Ratio of 1.45 [19]. On the other hand, a subgroup analysis of the Valsartan Antihypertensive Long‐term Use Evaluation (VALUE), which compared clinical outcomes of valsartan and amlodipine‐based treatment, reported no significant difference in terms of composite cardiac mortality and morbidity between these two treatment group regardless of ethnicity. However, because other antihypertensive medications were added to the treatment plan in most cases, these results could not be interpreted as the efficacy of ARB and CCBs in the monotherapy regimen [20, 21].

Our results also revealed lower odds of CVE diagnosis in patients using CCBs compared to diuretics in both Black and Caucasian cases. This finding, to a certain extent, is complementary to the findings of a large population cohort conducted in the United Kingdom, which reported that CCBs, compared to thiazides, were linked to greater reductions in systolic BP in all age groups of nonblack cases without diabetes at 12 weeks; however, this difference was diminished as the follow‐up duration increased, underscoring the importance of future long‐term investigations. However, a notable confidence interval overlap, in terms of BP reduction, was reported between these two drug classes in both Black and non‐Black cases [22]. On the other hand, and in contrast to our results, the ALLAHAT study [18] Reported a comparable rate of cardiovascular disorders between Amlodopine and chlorthalidone users in both Black and non‐Black subgroups. The difference between our findings and ALLAHAT could be attributed to the fact that we have included different types of medications of CCBs and diuretics while ALLAHAT only included amlodipine and chlorthalidone. This also agrees with the results of a propensity score‐matched cohort, that while CCBs demonstrated a similar BP reduction compared to thiazide‐like (such as chlorthalidone) drugs, these were more effective than older types of thiazides [22]. Furthermore, a large population meta‐analysis Included five trials comparing diuretics and CCBs that reported a lower risk of major cardiovascular events in diuretic groups [23]. However, these two groups were comparable for all types of events except for congestive heart failure. It should be noted that the result of this meta‐analysis could be confounded by the fact that in most of the included trials, using other antihypertensive agents was also allowed, which indicates that this could not be the certain result of comparing CCBs and diuretics efficacy as monotherapy agents in terms of CVE incidence, Which highlights the importance of our results in addressing this issue and clarifying the clinical outcomes of these drugs in each racial subgroups.

Considering both diuretics and CCBs, our results demonstrated comparable odds of being diagnosed with CVE in White versus Black populations. While the focus of our study is to compare the clinical outcomes of different types of antihypertensive drugs, some previous large population studies compared these drugs in terms of efficacy in blood pressure reduction, as a cohort of almost 10,000 hypertension cases conducted by T. Nguyen et al. [24] Demonstrated similar efficacy of CCBs in reaching target SBP and DBP in Blacks and non‐Blacks. Notably, a post hoc analysis of the PEAR‐2 trial illustrated a significant difference among different thiazides regarding reaching target BP in the White population, while these drugs were comparable in Blacks. Although the effectiveness of diuretics has been observed in both Whites and Blacks, This post hoc analysis highlights the importance of choosing the most appropriate diuretic agent for White patients [25].

Additionally, our findings suggest that White patients using ACE/ARBs were less likely to be diagnosed with a CVE in comparison to Black cases. This supports, to some extent, the findings of a cohort study of almost 59,000 hypertension patients [26], which illustrated a higher relative risk of composite cardiac adverse outcomes (including all‐cause mortality, acute MI, and stroke) of ACE to non‐ACE users in Blacks compared to Whites. This study, in line with our results, reported a significant interaction between race and type of hypertension treatment with a hazard ratio of 1.18, indicating a higher event rate in Blacks in comparison to whites. These findings are, to some extent, complementary to the results of previous studies, showing a higher efficacy of ACE/ARB in Blood pressure reduction in Whites compared to Blacks and indicating that this higher efficacy in BP control can also be observed in terms of the likelihood of CVE diagnosis [27, 28, 29]. However, the VALIANT trial results, which included 3730 patients with acute MI and a history of HF or left ventricular systolic dysfunction, demonstrated comparable clinical outcomes between African Americans and White Americans using valsartan or/and captopril [30]. These underscore the importance of future investigations on different clinical subpopulations of each racial group, as poorer outcomes that are observed in the overall ACE/ARB Black users compared to the White population should not be expected in all clinical subpopulations. In addition, it should be noted that Seghal et al. [31] reported that despite the considerable difference in Blood Pressure reduction, there is a > 80% overlap between the total population of different racial subgroups in terms of the value of SBP and DBP changes, which highlights the importance of considering other factors, as well as race, to optimize the treatment plan for each individual.

Our investigation revealed lower odds of CVE diagnosis in patients treated with ACE/ARBs compared to diuretic user cases in both White and Black populations. This superior outcome of ACE/ARB compared to diuretics is in contrast to the findings of the ALLHAT trial [18], which reported a higher risk of cardiovascular disease incidence in patients using ACEi compared to diuretics in both Black and non‐Black subgroups. Similarly, a propensity score‐matched cohort of almost 9000 hypertension cases demonstrated a higher rate of total CVE in HTN Black patients treated with ACEi compared to diuretics [19].

The contrast between our findings (lower CVE diagnosis likelihood in ACE/ARBS compared to diuretics) and the trials mentioned above [18, 19] could be further explained by the fact that these trials excluded patients with previous congestive heart failure and nonfatal myocardial infarction, which were not excluded in our studies. As stated in this study, these complications are the notable indications of ACE/ARBs [19]; furthermore, ACE/ARBs’ protective effect against adverse cardiac outcomes in heart failure cases has been shown in both African Americans and Caucasians [32]. Accordingly, the exclusion of heart failure cases may lead to diminished relative outcomes of ACE/ARBs in comparison to diuretics. Our findings highlight the importance of reconsidering current guidelines [8, 33] in terms of the indication and efficacy of ACE/ARBs in Black populations and their probable superiority over diuretic outcomes. These results suggest the possible benefits of these agents in specific Black as well as Caucasian subpopulations and the necessity of conducting future studies investigating precise predictive socioeconomic, genetic, and environmental factors for treatment responses, as there is still a lack of sufficient evidence regarding this issue [34, 35].

The overall rate of CVE, mortality, ICU admission, and LOS appeared to be higher in Black subgroups, while hospice discharge tended to be higher in White patients. These overall worse outcomes in the Black population, as well as baseline complications including DM and CKD are in agreement with the findings of previous population‐based studies [36, 37, 38]. This further highlights the importance of taking population‐based action to control the prevalence of risk factors and the increased rate of adverse outcomes in the Black hypertension population [35, 39].

## 6 Limitations and Strengths

The limitations of this study are as follows: First, it should be noted that the results of this study may have been confounded according to its retrospective design, and further prospective studies are needed to investigate the generalizability of our findings. Second, we excluded patients treated with more than one class of hypertension drugs. However, a growing body of literature suggests investigating the probable efficacy of treatment with more than one class of antihypertension drugs as a first‐line plan, which should be the focus of future large population studies. Third, we did not investigate the effect of the treatment dose for each drug, which should be considered in future investigations to determine the effect of dose in each drug class and ethnic subgroup. Fourth, we did not have information on the rate of controlled hypertension in each treatment subgroup. Furthermore, we did not have sufficient data on the patient's compliance with the treatment plan in each medication and racial subgroup. These should be noted when interpreting the results of this study. Fifth, If patients were taking more than 1 antihypertensive, they were excluded unless the second medication was an ACE or ARBs, in which case, they were included under the ACE/ARBs antihypertensive. This overinflated the number of patients taking ACE or ARBs, which could have biased the results. Sixth, Patients taking calcium channel inhibitors or diuretics could have been taking other antihypertensives that were not looked into in this study, such as alpha or beta blockers.

However, the strength of our study is noteworthy as a large sample size of both Blacks and non‐Hispanic Caucasians has been analyzed to increase the reliability and validity of the results. In addition, the effect of subpopulation with potentially confounding clinical situations has been minimized by determining appropriate exclusion criteria. Furthermore, subpopulations such as diabetic patients or heart failure cases that have been excluded in some of the previous studies are included in this investigation, causing our results to reflect the probable role of these clinical conditions in the efficacy of antihypertension drugs. This study is a registry‐based investigation that reflects the accuracy and reliability of the data used for analysis.

## 7 Conclusion

In conclusion, our study demonstrated that in both groups of CCBs and Diuretic users, the likelihood of being diagnosed with cardiovascular events is comparable between hypertensive African American and non‐Hispanic Caucasians. However, for ACE/ARB users, a significantly lower odd CVE diagnosis was observed in non‐Hispanic Caucasians compared to African American. Furthermore, in both racial subgroups of this study, taking CCBs before admission was associated with lower odds of CVE diagnosis compared to both diuretics or ACE/ARBs users. Furthermore, taking ACE/ARBs was associated with significantly lower odds of being diagnosed with CVE in both ethnical subgroups compared to diuretics.

## Author Contributions

A.H., S.R., and F.S. designed and drafted the manuscript. A.H., S.R., K.H., and F.S. contributed to the conception of the work and critically revised the manuscript. M.M. and T.S. conducted the statistical analysis. S.R. finalized the graphical abstract. A.H., S.R., F.H., and K.H. critically revised the manuscript. All authors gave final approval and agree to be accountable for all aspects of the work, ensuring its integrity and accuracy.

## Ethics Statement

Ethical approval for this study was obtained from the Institutional Review Board (IRB) of HCA Healthcare, under C.A.R.R.I.E. Submission ID #: 2023‐228.

## Consent

All authors, including the corresponding author, Sushil Rayamajhi, consent to the publication of this manuscript.

## Conflicts of Interest

This research was supported in whole or in part by HCA Healthcare and/or an HCA Healthcare‐affiliated entity. The views expressed in this publication represent those of the authors and do not necessarily reflect the official views of HCA Healthcare or any of its affiliated entities.

## Supporting information

Supporting information.

## Data Availability

The data that support the findings of this study are available from the corresponding author upon reasonable request. The data underlying this article will be shared upon reasonable request to the corresponding author.
