# Supplementary material for: Comparative Effectiveness of Calcium‐Channel Blockers, Angiotensin‐Converting Enzyme/Angiotensin Receptor Blockers and Diuretics on Cardiovascular Events Likelihood in Hypertensive African‐American and Non‐Hispanic Caucasians: A Retrospective Study Across HCA Healthcare
Source: Clin Cardiol. 2025 Jan 21;48(1):e70075. doi: 10.1002/clc.70075 (PMC11747351; doi:10.1002/clc.70075)
Supplement: Supplementary file 1 — Supporting information. [file CLC-48-e70075-s001.docx]

**SUPPLEMENTARY MATERIAL**

| **Table S1.** Medication Prior to Admission | | | | | | |
| --- | --- | --- | --- | --- | --- | --- |
| **Variables** | **Black** | | **Caucasian** | | **Overall** | |
|  | **n** | **%** | **n** | **%** | **n** | **%** |
| n, % | 3,404 | 22.9% | 11,441 | 77.1% | 14,845 | 100.0% |
| Statin | 1,362 | 40.0 | 5,325 | 46.5 | 6,687 | 45.0 |
| Aspirin | 1,002 | 29.4 | 3,609 | 31.5 | 4,611 | 31.1 |
| ACE/ARBSs | 1,965 | 57.7 | 7,682 | 67.1 | 9,647 | 65.0 |
| Calcium Blockers | 970 | 28.5 | 2,094 | 18.3 | 3,064 | 20.6 |
| Diuretics | 469 | 13.8 | 1,665 | 14.6 | 2,134 | 14.4 |

| **Table S2.** Association of Predicted Probabilities and Observed Responses | | | |
| --- | --- | --- | --- |
| **Percent Concordant** | **65.9** | **Somers' D** | **0.319** |
| **Percent Discordant** | **34.0** | **Gamma** | **0.319** |
| **Percent Tied** | **0.0** | **Tau-a** | **0.141** |
| **Pairs** | **48580203** | **c** | **0.660** |
